# Multiconfigurational Character of Repulsive A^2^Σ_g_^+^ State Leaves Strong Signature in the Photodissociation Spectrum of Zn_2_^+^

**DOI:** 10.1021/jacs.4c05620

**Published:** 2024-06-05

**Authors:** Dominik Jank, Milan Ončák, Shan Jin, Christian van der Linde, Martin K. Beyer

**Affiliations:** Institut für Ionenphysik und Angewandte Physik, Universität Innsbruck, Technikerstraße 25, 6020 Innsbruck, Austria

## Abstract

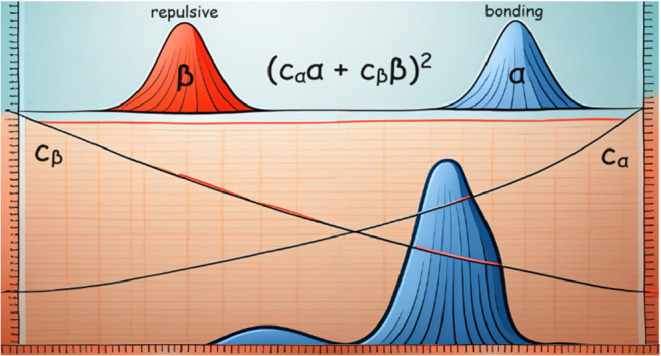

For the excitation to a repulsive state of a diatomic molecule, one expects a single broad peak in the photodissociation spectrum. For Zn_2_^+^, however, two peaks for the spin- and symmetry-allowed A^2^Σ_g_^+^ ← X^2^Σ_u_^+^ transition are observed. A detailed quantum-chemical analysis reveals pronounced multiconfigurational character of the A^2^Σ_g_^+^ state. The σ_g_(4s)^2^σ_g_(4p) configuration with bond order 1.5 dominates at short distances, while the repulsive σ_g_(4s)σ_u_^*^(4s)^2^ configuration with bond order −0.5 wins over with increasing bond length. The two excited-state configurations contribute with opposite signs to the transition dipole moment, which reaches zero near the equilibrium distance. This local minimum of the oscillator strength is responsible for the pronounced dip in the photodissociation spectrum, which is thus the spectroscopic signature of the multiconfigurational character of the A^2^Σ_g_^+^ state.

Metal dimers have been studied extensively as model systems for chemical bonding.^1^ They exhibit a wide range of bonding patterns, from the weak van der Waals bond found in Zn_2_ with a formal bond order of zero^2,3^ to strong covalent bonding in Cr_2_ with a formal bond order of 6.^4−7^ Despite the small polarizability of beryllium atoms, Be_2_ is significantly more strongly bound than Zn_2_, with spectroscopic constants *D*_e_(Be_2_) = 930 ± 2 cm^–1^ versus *D*_e_(Zn_2_) = 215 ± 1 cm^–1^.^3,8^ This somewhat surprising experimental result is due to the multiconfigurational character of the electronic wave function in both molecules and subtle stabilizing and destabilizing effects, which have been analyzed and documented in numerous publications, mostly on Be_2_.^8,9^ Despite its success in describing relative energies of electronic states and predicting bond lengths, the concept of multiconfigurational wave functions^10,11^ remains elusive, since their existence is mainly deduced from quantum-chemical calculations. Electronic absorption spectra by themselves do not reveal directly whether a multiconfigurational wave function is involved.

Upon removal of an electron, the resulting Be_2_^+^ has a formal bond order of 0.5 and a ^2^Σ_u_^+^ ground state.^12,13^ No experimental spectroscopic data are available for Zn_2_^+^. Gutsev and Bauschlicher showed with density functional theory (DFT) calculations that the dimer has a filled σ_g_(4s) orbital and a half-filled σ_u_^*^(4s) orbital,^14^ resulting in a bond order of 0.5 for the ^2^Σ_u_^+^ ground state, as illustrated in Scheme 1. Two excited-state configurations with ^2^Σ_g_^+^ symmetry can be reached by exciting either an α or β electron. In the β-electron-excited configuration σ_g_(4s)σ_u_^*^(4s)^2^, the formal bond order is reduced to −0.5, while the α-electron-excited configuration σ_g_(4s)^2^σ_g_(4p) results in a formal bond order of 1.5.

**Scheme 1:**
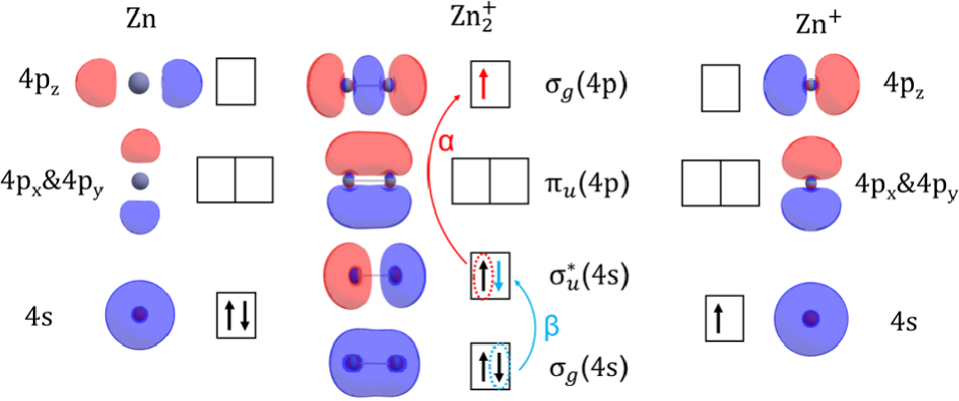
Molecular Orbital Diagram of Zn_2_^+^ Electrons in the ^2^Σ_u_^+^ ground-state configuration are shown with black arrows. Excitation of an α (red) or β (blue) electron results in a ^2^Σ_g_^+^ excited state.

We recently performed infrared multiple photon dissociation (IRMPD) spectroscopy of hydrated Zn_2_^+^(H_2_O)_*n*_ in the gas phase,^15^ showing that the zinc dimer resides on the surface of the water clusters, similar to Zn^+^(H_2_O)_*n*_.^16,17^ Here we examine the stability and electronic structure of the unsolvated Zn_2_^+^ dimer by photodissociation spectroscopy. The simple molecular orbital (MO) picture in Scheme 1 suggests that excitation to the repulsive σ_g_(4s)σ_u_^*^(4s)^2^ configuration would result in one broad peak in the photodissociation spectrum, characteristic of excitation to a dissociative state in diatomic molecules. However, we observe a pronounced dip in the broad peak, which is caused by the multiconfigurational character of the repulsive A^2^Σ_g_^+^ state.

Photodissociation spectroscopy was performed on a 4.7 T FT-ICR mass spectrometer,^18,19^ with Zn_2_^+^ ions obtained by laser vaporization,^20−22^ using a tunable pulsed OPO laser system for irradiation. Over the studied photon energy range of 1.9 to 5.5 eV, the photodissociation signal was observed mainly between 2.3 and 3.1 eV, with a prominent peak centered at 2.92 eV and a weaker one at 2.57 eV (see Figure 1). Weak features near the detection limit can also be found for energies above 4.5 eV, indicating a contribution of higher-lying excited states. The split peak cannot be caused by spin–orbit splitting, since Σ states do not split. Another common explanation would be the vibrational excitation of a fraction of the Zn_2_^+^ ions in the electronic ground state. However, the calculated vibrational excitation energy of Zn_2_^+^ is 20 meV, while the peak separation is 350 meV, a total mismatch.

**Figure 1:**
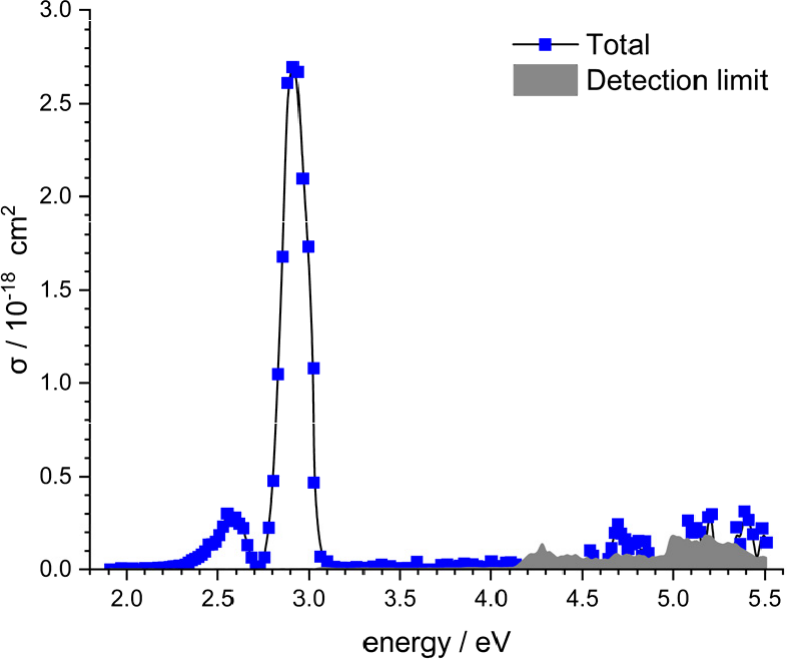
Experimental photodissociation spectrum of Zn_2_^+^.

To understand the reason for the split peak, we calculated the excited-state potential curves of Zn_2_^+^ at various levels of theory, as shown in Figure 2a. Coupled cluster singles, doubles, and noniteratively included triples (CCSD, CCSD(T)), equation of motion CCSD (EOM-CCSD), and time-dependent DFT (TDDFT) calculations were performed with Gaussian 16,^23^ and multireference configuration interaction (MRCI) calculations were carried out with MOLPRO.^24^ As expected from the MO picture in Scheme 1, a low-lying repulsive A^2^Σ_g_^+^ state is present. The lowest-lying bound excited state B^2^Π_u_ results from the parity-forbidden π_u_(4p) ← σ_u_^*^(4s) transition. Three higher-lying states are available in the studied energy range, but they only produce a weak photodissociation signal since excitation from the ground state is only allowed into the bound C^2^Σ_g_^+^ and D^2^Π_g_ states (see Table S3). Since the potential curves do not provide a pathway for dissociation in these states, the absorption of a second photon within the same laser pulse is the most plausible explanation for the observed weak photodissociation signal at higher energies. A contribution of the B^2^Π_u_ or E^2^Σ_u_^+^ state to the photodissociation signal can be ruled out, as the transition from the ground state is parity-forbidden and switching between the B^2^Π_u_ and A^2^Σ_g_^+^ potential curves is prohibited by their symmetry mismatch.

**Figure 2:**
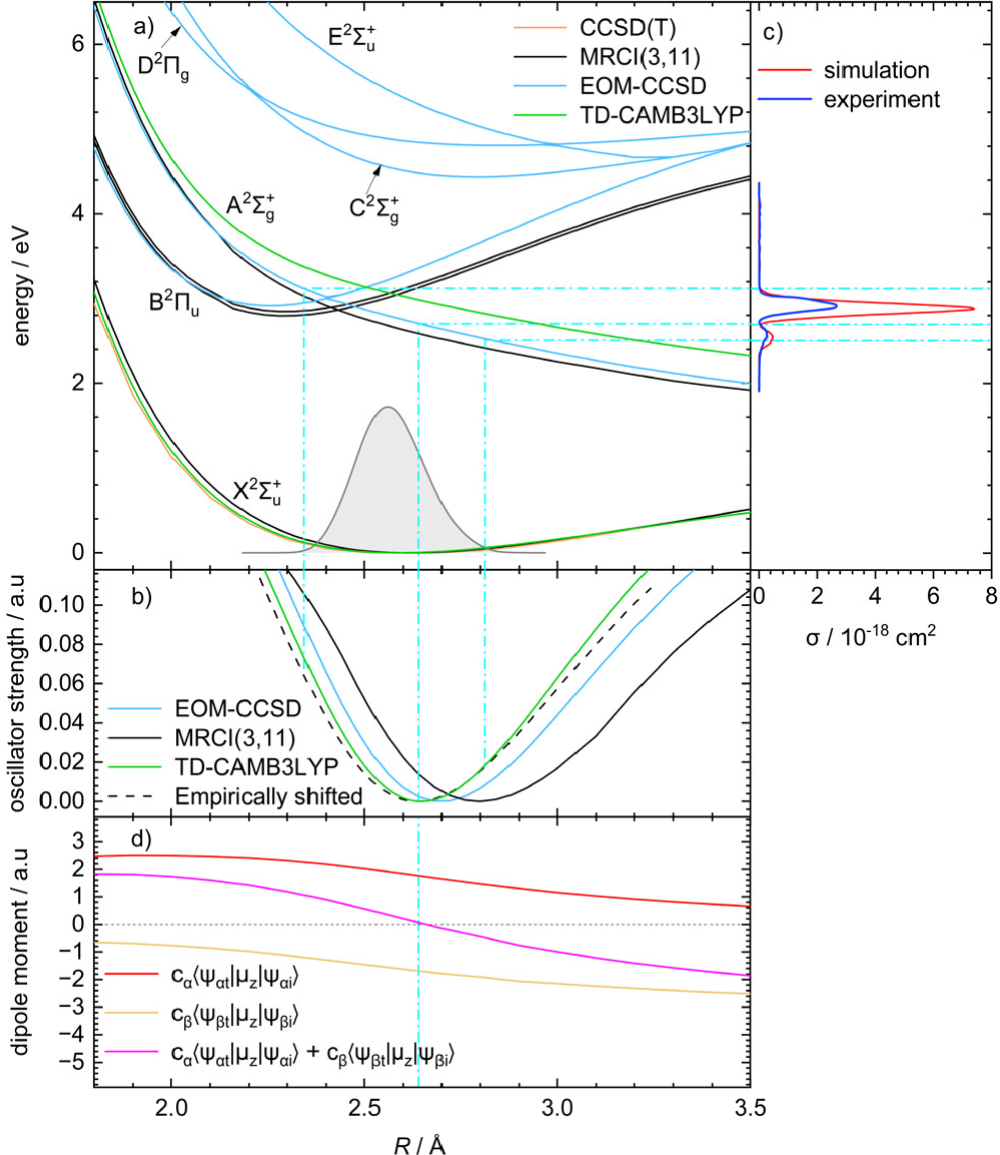
(a) Potential curves of the ground and first five excited states of Zn_2_^+^ at various levels of theory (MRCI/aug-cc-pVQZ, EOM-CCSD(full)/aug-cc-pVQZ, TD-CAMB3LYP/aug-cc-pVTZ, CCSD(T)/aug-cc-pVQZ). (b) Oscillator strength of the A^2^Σ_g_^+^ ← X^2^Σ_u_^+^ transition as a function of bond length *R* along with the empirically shifted EOM-CCSD curve. (c) Experimental spectrum and the simulated spectrum with the empirically shifted oscillator strength, the thermalized ground-state density, and the zero-point-corrected excitation energy. (d) Contributions of the α and β electron excitations and resulting transition dipole moment as calculated at the TD-CAMB3LYP/aug-cc-pVDZ level of theory.

To simulate the peak shape with the reflection principle,^25−27^ we calculated the transition dipole moment for the transition from the X^2^Σ_u_^+^ ground state to the A^2^Σ_g_^+^ excited state as a function of the Zn–Zn distance *R*, as shown in Figure 2d. On all levels of theory, the transition dipole moment consistently reaches zero near the equilibrium bond length of Zn_2_^+^ and rises toward the classical turning points of the zero-point vibration, with a steeper increase for the compressed molecule.

Using these results, we simulated the photodissociation spectrum, as shown in Figure 2c. The reflection principle requires a realistic ground-state vibrational density, which we modeled by solving the Schrödinger equation and accounting for the thermal population of the vibrational mode (see the Supporting Information (SI) for details). With the calculated transition dipole moments and excitation energies as a function of *R*, we indeed obtain the observed split peak as shown in Figure 2c (see the SI for additional details). The separation between the two peak maxima is reproduced faithfully on all theory levels; the position of the intensity minimum, however, deviates from experiment and is very sensitive to the theory level employed for the calculation of the oscillator strength. To reproduce the experiment, we shifted the minimum of the oscillator strength by 0.06 Å to smaller *R*, which translates to a shift of 0.04 eV in excitation energy, well below the expected error of the excited-state calculation.

To analyze the strong dependence of the oscillator strength on the Zn–Zn distance, we take a closer look at the character of the transitions and the involved MOs. Natural transition orbitals (NTOs) obtained by TDDFT are shown in Figure 3a, calculated at *R* = 2.65 Å, close to the oscillator strength minimum. Consistent with EOM-CCSD as well as MRCI calculations, we find that both the α-electron-excited configuration σ_g_(4s)^2^σ_g_(4p) and the β-electron-excited configuration σ_g_(4s)σ_u_^*^(4s)^2^ shown in Scheme 1 contribute to the A^2^Σ_g_^+^ excited state, *i.e.*, the state has a pronounced multiconfigurational character. The coefficients listed in Table 1 show that the α-electron excitation dominates at short distances *R*, while the β-electron excitation takes over at longer *R*. This makes sense since a higher population of the bonding σ_g_(4p) MO is energetically favorable at short distances, while the antibonding σ_u_^*^(4s) MO is inherently lower in energy for higher values of *R* because the 4s atomic orbitals (AOs) lie energetically well below the 4p AOs. The higher-lying C^2^Σ_g_^+^ state corresponds to a complementary combination of the two configurations. Here, the repulsive β-electron excitation dominates at small *R*, while the attractive α-electron excitation turns C^2^Σ_g_^+^ into a bound state at large *R*.

**Figure 3:**
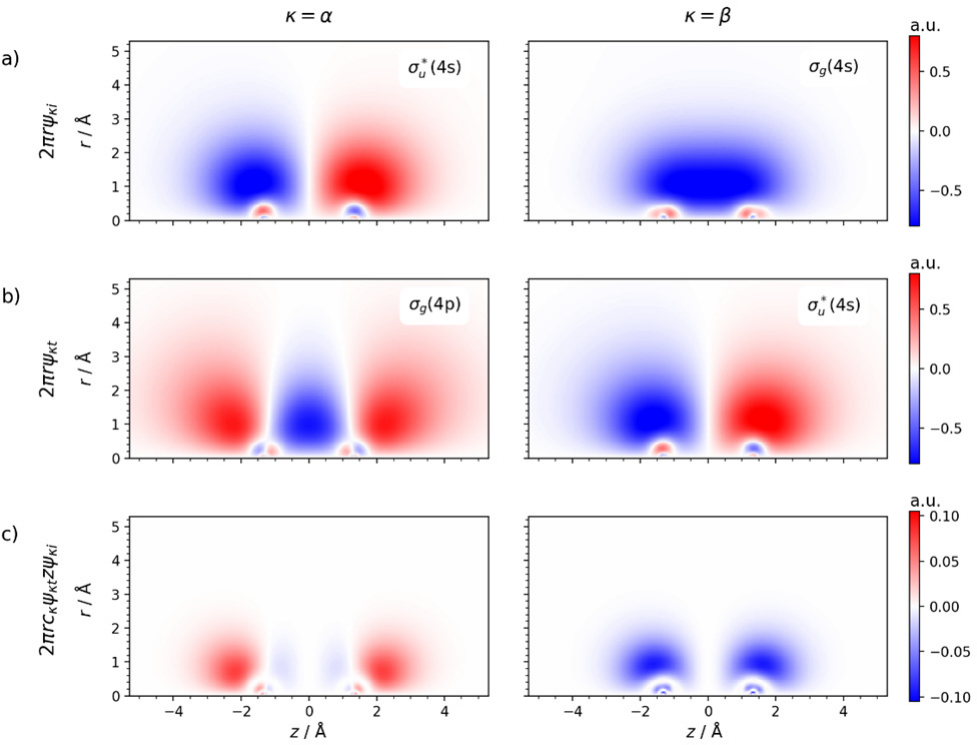
Illustration of the contributions of α- and β-electron excitations to the transition dipole moment, based on NTOs of Zn_2_^+^ calculated at the TD-CAMB3LYP/aug-cc-pVDZ level of theory with *R* = 2.65 Å. (a) 2π*r*ψ_κi_ and (b) 2π*r*ψ_κt_ (κ = α, β) correspond closely to the MOs from Scheme 1. (c) Local contributions to the transition dipole moment 2π*rc*_κ_ψ_κt_*zψ*_κi_. The molecular axis corresponds to the *z* coordinate with the origin placed at the center of inversion.  is the distance from the molecular axis.

**Table 1:** Contributions *c*_α_ and *c*_β_ of the α- and β-Electron Transition Dipole Moments μ_*z*,α_ = ⟨ψ_αt_|μ_*z*_|ψ_αi_⟩ and μ_*z*,β_ = ⟨ψ_βt_|μ_*z*_|*ψ*_βi_⟩ (in a.u.) to the Oscillator Strength *f*_it_ at Various Zn–Zn Distances *R* (in Å)^a^

*R*	*c*_α_	*c*_β_	μ_*z*,α_	μ_*z*,β_	*f*_TD_	*f*_EOM_
1.80	0.93	0.44	2.66	–1.47	0.25	0.26
2.30	0.85	0.65	2.71	–1.74	0.09	0.10
2.65	0.66	0.84	2.63	–2.03	0.00	0.00
3.20	0.38	0.90	2.40	–2.58	0.11	0.09
4.20	0.16	0.74	2.01	–3.68	0.17	0.17

a The values were calculated at the TD-CAMB3LYP/aug-cc-pVDZ level of theory. The oscillator strength is also provided at the EOMCCSD(full)/aug-cc-pVQZ level (*f*_TD_ and *f*_EOM_, respectively).

In the next step, we link the multiconfigurational character of the A^2^Σ_g_^+^ state to the minimum of the oscillator strength. Neglecting small contributions from other configurations, we obtain the oscillator strength *f*_it_ between initial state i and target state t from eq 1:
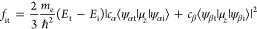
1where *m*_e_ is the electron mass, *E*_t_ – *E*_i_ is the excitation energy, and *c*_α_ and *c*_β_ are the coefficients of the α- and β-electron excitations given in Table 1, which depend strongly on *R*. ⟨ψ_κt_|μ_*z*_|ψ_κi_⟩ are transition dipole moments with the ground-state (initial) wave functions ψ_αi_ and ψ_βi_ and excited-state (target) wave functions ψ_αt_ and ψ_βt_. As the transition dipole moments for the α- and β-electron transitions are positive and negative, respectively, for all *R*, the sum necessarily changes sign while going from short to long *R*, reaching zero in between (see Table 1 and Figure 2d).

Figure 3 illustrates why the transition dipole moments of the α- and β-electron transitions have different sign. The α-electron transition dipole moment is dominated by the positive lobes of the σ_g_(4p) target MO (Figure 3b), while the sign of the β-electron transition dipole moment is determined by the mostly negative σ_g_(4s) initial MO (Figure 3a). In other words, the different phases of these two MOs in the regions that contribute most to the transition dipole moment are responsible for the different signs and cause the annihilation of the oscillator strength at a specific value of *R*.

The mixing of the σ_g_(4s)σ_u_^*^(4s)^2^ and σ_g_(4s)^2^σ_g_(4p) configurations that dominate the A^2^Σ_g_^+^ state of Zn_2_^+^ is reminiscent of the multiconfigurational ground state of the intensively studied beryllium dimer Be_2_.^8^ In both cases, the difference in energy between the outer s and p orbitals is very small.^28^ For Be_2_, this affects the X^2^Σ_u_^+^ ground state, while for Zn_2_^+^, we see the effect in the A^2^Σ_g_^+^ excited state. The Be dimer, a seemingly simple system with only eight electrons, has posed considerable challenges for quantum chemistry. Up to its first experimental characterization by Bondybey and English,^29^ it was long a mystery whether it was a bound system at all.^30^ Interestingly, a strongly distance-dependent oscillator strength was predicted in a theoretical study of Be_2_^+^,^12^ also for the A^2^Σ_g_^+^ ← X^2^Σ_u_^+^ transition. Despite the multiconfigurational character of the A^2^Σ_g_^+^ state of Zn_2_^+^, our results are quite robust with respect to the theory level. All calculations, including TDDFT, reproduce the mixing of the configurations (also see the SI). The deviations in energy and structure are in the typical ranges for excited-state calculations.

The photodissociation spectrum of the zinc dimer cation exhibits two well-separated peak maxima, which arise from electronic excitation to the same repulsive state, A^2^Σ_g_^+^. The reason for the local disappearance of absorption intensity lies in the strong dependence of the transition dipole moment on the Zn–Zn bond length, which in turn arises from the multiconfigurational character of the A^2^Σ_g_^+^ state. The resulting dip in the photodissociation peak is the unique signature of the multiconfigurational wave function of the A^2^Σ_g_^+^ state. It may serve as a sensitive experimental benchmark for the construction of multiconfigurational wave functions, since the position of the dip relies on the delicate balance of the contributing configurations.

## Supporting Information Available

Computational details and benchmarks (Figures S1–S4 and Tables S1–S3) and experimental data from Figure 1 (PDF)

## References

[ref1] Morse M. D. Clusters of transition-metal atoms. Chem. Rev. 1986, 86, 1049–1109. 10.1021/cr00076a005.

[ref2] Czuchaj E.; Rebentrost F.; Stoll H.; Preuss H. Potential energy curves for the Zn_2_ dimer. Chem. Phys. Lett. 1996, 255, 203–209. 10.1016/0009-2614(96)00336-3.

[ref3] Czajkowski M.; Bobkowski R.; Krause L. O_u_^+^(^3^Π_u_)←XO_g_^+^(^1^Σ_g_^+^) transitions in Zn_2_ excited in crossed molecular and laser beams. Phys. Rev. A 1990, 41, 277–282. 10.1103/PhysRevA.41.277. 9902868

[ref4] Larsson H. R.; Zhai H.; Umrigar C. J.; Chan G. K.-L. The Chromium Dimer: Closing a Chapter of Quantum Chemistry. J. Am. Chem. Soc. 2022, 144, 15932–15937. 10.1021/jacs.2c06357. 36001866 PMC9460780

[ref5] Casey S. M.; Leopold D. G. Negative ion photoelectron spectroscopy of chromium dimer. J. Phys. Chem. 1993, 97, 816–830. 10.1021/j100106a005.

[ref6] Bondybey V. E.; English J. H. Electronic structure and vibrational frequency of Cr_2_. Chem. Phys. Lett. 1983, 94, 443–447. 10.1016/0009-2614(83)85029-5.

[ref7] Kraus D.; Saykally R. J.; Bondybey V. E. Cavity ringdown spectroscopy search for transition metal dimers. Chem. Phys. 1999, 247, 431–434. 10.1016/S0301-0104(99)00210-4.

[ref8] Merritt J. M.; Bondybey V. E.; Heaven M. C. Beryllium dimer—caught in the act of bonding. Science 2009, 324, 1548–1551. 10.1126/science.1174326. 19460963

[ref9] Heaven M. C.; Merritt J. M.; Bondybey V. E. Bonding in beryllium clusters. Annu. Rev. Phys. Chem. 2011, 62, 375–393. 10.1146/annurev-physchem-032210-102545. 21219142

[ref10] Plasser F.; Mewes S. A.; Dreuw A.; González L. Detailed Wave Function Analysis for Multireference Methods: Implementation in the Molcas Program Package and Applications to Tetracene. J. Chem. Theory Comput. 2017, 13, 5343–5353. 10.1021/acs.jctc.7b00718. 28972759

[ref11] Ghosh S.; Verma P.; Cramer C. J.; Gagliardi L.; Truhlar D. G. Combining Wave Function Methods with Density Functional Theory for Excited States. Chem. Rev. 2018, 118, 7249–7292. 10.1021/acs.chemrev.8b00193. 30044618

[ref12] Fischer I.; Bondybey V. E.; Rosmus P.; Werner H.-J. Theoretical study of the electronic states of BeLi and Be_2_^+^. Chem. Phys. 1991, 151, 295–308. 10.1016/0301-0104(91)80016-B.

[ref13] Merritt J. M.; Kaledin A. L.; Bondybey V. E.; Heaven M. C. The ionization energy of Be_2_, and spectroscopic characterization of the (1)^3^Σ_u_^+^, (2)^3^Π_g_, and (3)^3^Π_g_ states. Phys. Chem. Chem. Phys. 2008, 10, 4006–4013. 10.1039/b803975e. 18597014

[ref14] Gutsev G. L.; Bauschlicher C. W. Chemical Bonding, Electron Affinity, and Ionization Energies of the Homonuclear 3d Metal Dimers. J. Phys. Chem. A 2003, 107, 4755–4767. 10.1021/jp030146v.

[ref15] Cunningham E. M.; Taxer T.; Heller J.; Ončák M.; van der Linde C.; Beyer M. K. Asymmetric Solvation of the Zinc Dimer Cation Revealed by Infrared Multiple Photon Dissociation Spectroscopy of Zn_2_^+^(H_2_O)_*n*_ (*n* = 1–20). Int. J. Mol. Sci. 2021, 22, 6026 10.3390/ijms22116026. 34199627 PMC8199724

[ref16] Bandyopadhyay B.; Reishus K. N.; Duncan M. A. Infrared Spectroscopy of Solvation in Small Zn^+^(H_2_O)_*n*_ Complexes. J. Phys. Chem. A 2013, 117, 7794–7803. 10.1021/jp4046676. 23875934

[ref17] Cunningham E. M.; Taxer T.; Heller J.; Ončák M.; van der Linde C.; Beyer M. K. Microsolvation of Zn Cations: Infrared Multiple Photon Dissociation Spectroscopy of Zn^+^(H_2_O)_*n*_ (*n* = 2–35). Phys. Chem. Chem. Phys. 2021, 23, 3627–3636. 10.1039/D0CP06112C. 33524092

[ref18] Berg C.; Schindler T.; Niedner-Schatteburg G.; Bondybey V. E. Reactions of Simple Hydrocarbons with Nb_*n*_^+^: Chemisorption and Physisorption on Ionized Niobium Clusters. J. Chem. Phys. 1995, 102, 4870–4884. 10.1063/1.469535.

[ref19] Höckendorf R. F.; Balaj O. P.; van der Linde C.; Beyer M. K. Thermochemistry from ion–molecule reactions of hydrated ions in the gas phase: a new variant of nanocalorimetry reveals product energy partitioning. Phys. Chem. Chem. Phys. 2010, 12, 3772–3779. 10.1039/b921395c. 20358037

[ref20] Bondybey V. E.; English J. H. Laser Induced Fluorescence of Metal Clusters Produced by Laser Vaporization: Gas Phase Spectrum of Pb_2_. J. Chem. Phys. 1981, 74, 6978–6979. 10.1063/1.441064.

[ref21] Dietz T. G.; Duncan M. A.; Powers D. E.; Smalley R. E. Laser Production of Supersonic Metal Cluster Beams. J. Chem. Phys. 1981, 74, 6511–6512. 10.1063/1.440991.

[ref22] Duncan M. A. Invited Review Article: Laser Vaporization Cluster Sources. Rev. Sci. Instrum. 2012, 83, 041101 10.1063/1.3697599. 22559508

[ref23] Frisch M. J.; Trucks G. W.; Schlegel H. B.; Scuseria G. E.; Robb M. A.; Cheeseman J. R.; Scalmani G.; Barone V.; Petersson G. A.; Nakatsuji H.; Gaussian 16, rev. A.03; Gaussian, Inc.: Wallingford, CT, 2016.

[ref24] Werner H.-J.; Knowles P. J.; Knizia G.; Manby F. R.; Schütz M. Molpro: a general-purpose quantum chemistry program package. Wiley Interdiscip. Rev.: Comput. Mol. Sci. 2012, 2, 242–253. 10.1002/wcms.82.

[ref25] Lee S. Y.; Brown R. C.; Heller E. J. Multidimensional reflection approximation: Application to the photodissociation of polyatomics. J. Phys. Chem. 1983, 87, 2045–2053. 10.1021/j100235a006.

[ref26] Prakash M. K.; Weibel J. D.; Marcus R. A. Isotopomer fractionation in the UV photolysis of N_2_O: Comparison of theory and experiment. J. Geophys. Res.: Atmos. 2005, 110, D21315 10.1029/2005JD006127.

[ref27] Ončák M.; Šištík L.; Slavíček P. Can theory quantitatively model stratospheric photolysis? Ab initio estimate of absolute absorption cross sections of ClOOCl. J. Chem. Phys. 2010, 133, 174303 10.1063/1.3499599. 21054028

[ref28] El Khatib M.; Bendazzoli G. L.; Evangelisti S.; Helal W.; Leininger T.; Tenti L.; Angeli C. Beryllium dimer: a bond based on non-dynamical correlation. J. Phys. Chem. A 2014, 118, 6664–6673. 10.1021/jp503145u. 24866399

[ref29] Bondybey V. E.; English J. H. Laser vaporization of beryllium: Gas phase spectrum and molecular potential of Be_2_. J. Chem. Phys. 1984, 80, 568–570. 10.1063/1.446434.

[ref30] Liu B.; McLean A. D. Ab initio potential curve for Be_2_(^1^Σ_g_^+^) from the interacting correlated fragments method. J. Chem. Phys. 1980, 72, 3418–3419. 10.1063/1.439528.

