# Supplementary material for: Multiconfigurational Character of Repulsive A2Σg+ State Leaves Strong Signature in the Photodissociation Spectrum of Zn2+
Source: J Am Chem Soc. 2024 Jun 5;146(24):16385–8. doi: 10.1021/jacs.4c05620 (PMC11191677; doi:10.1021/jacs.4c05620)
Supplement: Supplementary file 1 — ja4c05620_si_001.pdf [file ja4c05620_si_001.pdf]

Supporting Information: Multiconfigurational Character of Repulsive $A^2\Sigma_g^+$ State Leaves Strong Signature in the Photodissociation Spectrum of Zn_2^+

Dominik Jank, Milan Ončák,* Shan Jin, Christian van der Linde, Martin K. Beyer*

Institut für Ionenphysik und Angewandte Physik, Universität Innsbruck, Technikerstraße 25, 6020 Innsbruck, Austria

Email: milan.oncak@uibk.ac.at; martin.beyer@uibk.ac.at

Computational details

The potential curve of the electronic ground state of Zn_2^+ was calculated at the CCSD(T)/aug-cc-pVQZ level of theory. The coupled cluster approach was used both with freezing the core electrons and without ("full"). A benchmark with CCSD and CCSD(T) using aug-cc-pV*X*Z basis sets, with *X* ranging from double to quadruple zeta, frozen as well as unfrozen core electrons and multi-reference methods validates the used method, see Table S1.

For excited state modeling, Equation of Motion Coupled Clusters Singles and Doubles (EOM-CCSD), Multi-Reference Configuration Interaction (MRCI), and time-dependent density functional theory (TDDFT) calculations we used, with the aug-cc-pVQZ basis set, unfrozen core electrons for EOM-CCSD, a (3,11) active space for the MRCI calculations where also spin orbit (SO) coupling is considered. SO coupling has only a small influence on the energy as the Σ state cannot split. The CAM-B3LYP functional is employed for TDDFT calculations. Benchmarks are performed by EOM-CCSD calculations using aug-cc-pV*X*Z, and aug-cc-pWCV*X*Z basis sets with *X* ranging from double to quadruple zeta with frozen as well as unfrozen core electrons and MRCI calculations with the same range of basis sets and active spaces of (3,5), (3,9) and (3,11), see Tables S1 and S2. Results agree for all methods and basis sets within the expected error limits. A small shift to larger *R* for the potential minima as well as the oscillator strength minima is obtained for MRCI calculations. For spectrum modeling, the target $A^2\Sigma_g^+$

state is described through single-point EOM-CCSD (full)/aug-cc-pVQZ calculations for *R* = 2–3.3 Å, at a step size of 0.005 Å.

To simulate the spectrum, we employ reflection principle. For the ground state density ρ_{GS}, we used the vibrational wave functions φ_v and energies E_v obtained through numerical solution of the Schrödinger equation on the CCSD(T)/aug-cc-pVQZ potential energy surface splined with 10,000 points for a smoother curve. The thermally distributed density for a single vibrational state *v* can then be calculated according to the Boltzmann equation

$$p = \frac{\exp\left(\frac{E_v}{k_{\mathrm{B}}T}\right)}{Z}$$

using the Boltzmann constant k_{B} and a temperature T = 300 K to match the experimental conditions. *Z* is the canonical partition function, in our case just the sum over all vibrational states. Taking into account only states up to *v* = 5 is sufficient for our case, as higher states do not contribute for these temperatures. Thus, we end up for the density of one vibrational state with:

$$\rho_v = \frac{\exp\left(\frac{E_v}{k_{\mathrm{B}}T}\right)}{\sum_v \exp\left(\frac{E_v}{k_{\mathrm{B}}T}\right)} |\varphi_v|^2$$

and the GS density follows with $\rho_{\mathrm{GS}} = \sum_v^5 \rho_v$, see Figure S4.

We calculate the intensity for a given excitation energy E_i, using energy bins with an interval of $[E_i, E_i + \Delta E_i]$, with different energy sizes ΔE_i but with continuous step size on the PES of 0.005 Å. Considering also the oscillator strength f_{it} we can calculate the intensity:

$$I_{\mathrm{MC},i} = \int_{E_i}^{E_i+\Delta E_i} dE\, f_{it}\rho_v$$

For conversion from intensity to the absolute absorption cross section, the following formula is used.[1]

$$\sigma(E_i) = \frac{\pi E_i I_{\mathrm{MC},i}}{3\hbar \mathcal{E}_0 c \Delta E_i}$$

Summing over the cross sections of all vibronic states gives the total cross section for a given excitation energy.

$$\sigma(E_{i,tot}) = \sum_{v} \sigma(E_v)$$

Visualization of the wave functions for the transitions was performed within an in-house code, using the output file of GAUSSIAN calculations, using the explicit form of Gaussian-type orbitals (GTOs):

$$\Phi(x, y, z, \alpha, , i, j, k) = \left(\frac{2\alpha}{\pi}\right)^{3/4} \left[\frac{(8\alpha)^{i+j+k} i! \, j! \, k!}{(2i)! \, (2j)! \, (2k)!}\right]^{1/2} x^i y^j z^k e^{-\alpha(x^2+y^2+z^2)}$$

With the coordinates x, y and z, α the width, and i, j and k are the quantum numbers of the spherical harmonics in the three Cartesian directions, respectively.

Atomic orbitals (AOs) are calculated numerically in a cubic grid of a size of 250x250x250 grid points and a box size of 20x20x20 Bohr. In the next step, molecular orbitals (MOs) for different electronic states were constructed. The coefficients of the AOs contributing to a MO are taken from the Gaussian output, and the MOs are simple linear combinations of the single AOs with the factors of the output for the corresponding atom then. The MO represents than the wave functions $\psi_{\kappa\zeta}$, for certain orbitals, κ denoting if the wave function corresponds to an excitation of an α or β electron, ζ if it is from the initial i or target t state. These wave functions were visualized and used to calculate matrix elements $\langle\psi_{\kappa t}|\mu_z|\psi_{\kappa i}\rangle$ and total transition dipole moment $c_\alpha\langle\psi_{\alpha t}|\mu_z|\psi_{\alpha i}\rangle + c_\beta\langle\psi_{\beta t}|\mu_z|\psi_{\beta i}\rangle$, which were compared to the output from Gaussian for verification purposes. For visualization, we chose a 2D representation. As electronic states of interest are all of Σ symmetry with cylindrical symmetric, there is a possibility of a loss-free 2D visualization along the symmetry axis, which is chosen as the molecular *z* axis in this case. We take the points in the *xz* plane of the cube and multiply the elements by $2\pi r$, with the distance r from the symmetry axis and corresponds to the integral over the spherical angle $\int_0^{2\pi} r d\varphi = 2\pi r$.

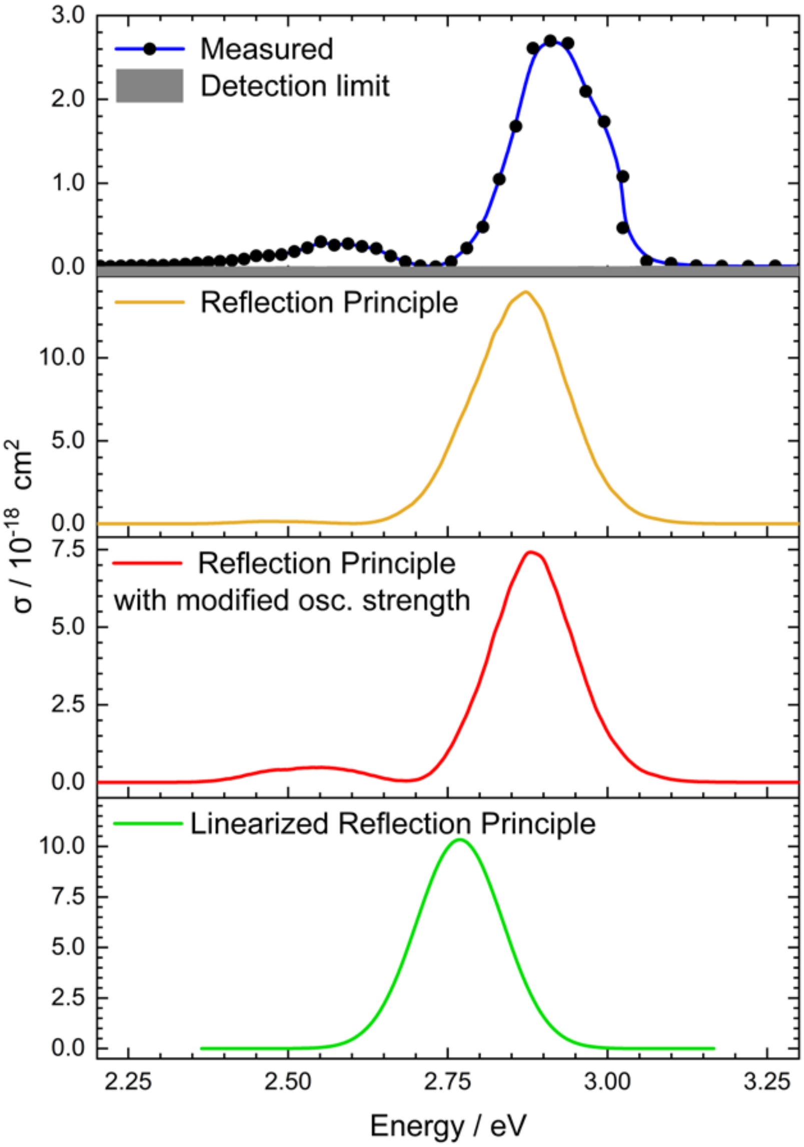

Figure S1: Experimental photodissociation spectrum of Zn_2^+ (blue); spectrum simulated with the reflection principle and *R*-dependent oscillator strength at 300K (yellow); simulated spectrum with oscillator strength shifted by 0.06 Å to lower *R* (red); spectrum simulated with the linearized reflection principle (LRP) (green). Using EOM-CCSD(full)/aug-cc-pVQZ level of theory for the excited states and CCSD(T)(full)/aug-cc-pVQZ for modeling the ground state density.

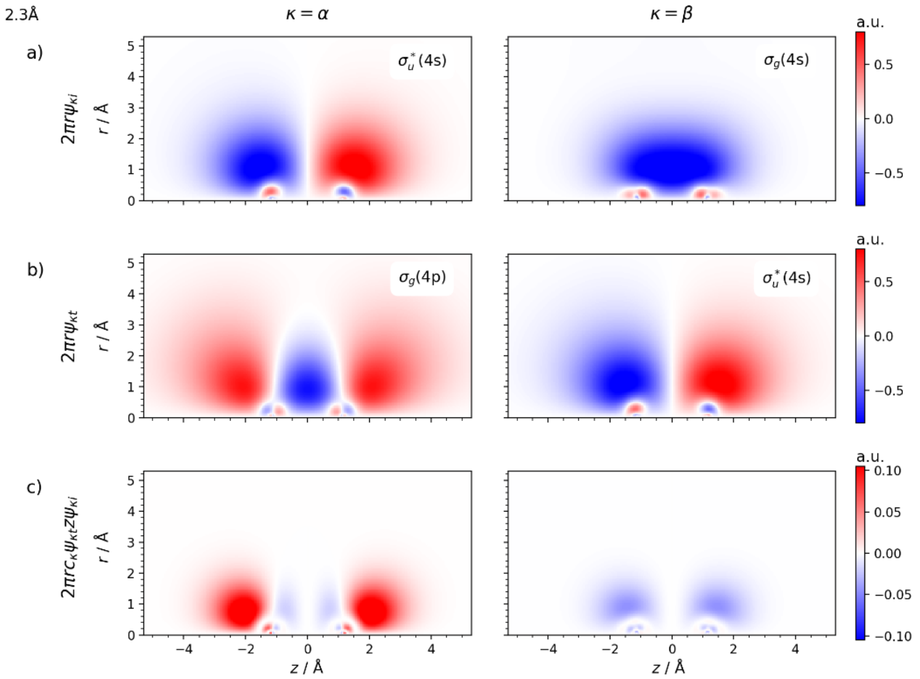

Figure S2: The same data as in Figure 3 of the main text, here for R = 2.3 Å.

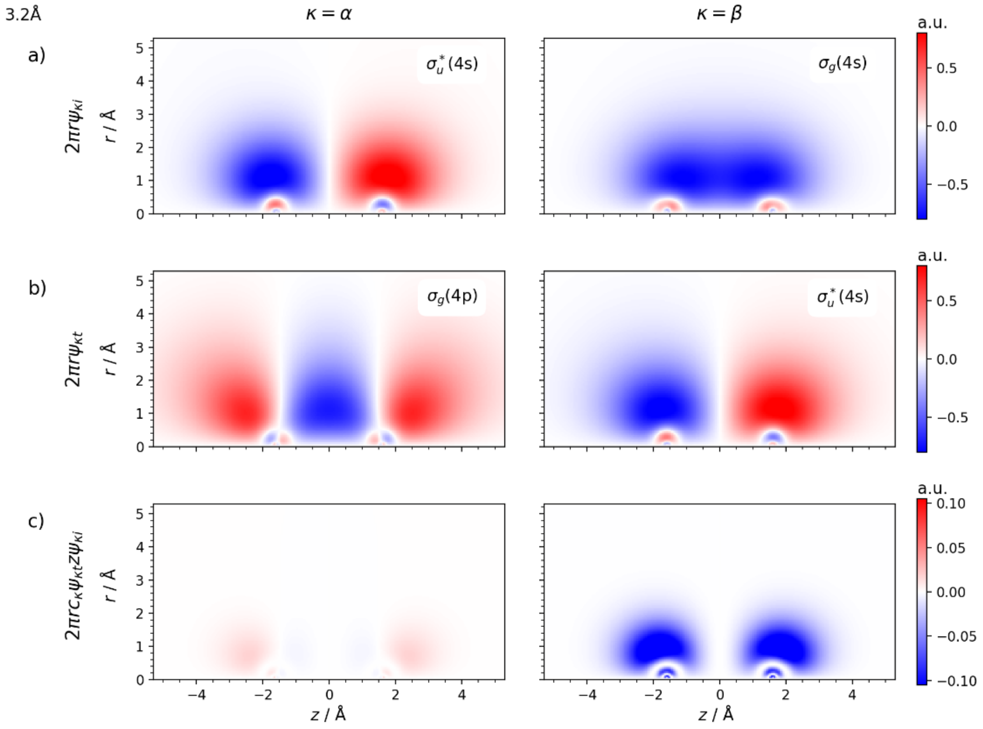

Figure S3: The same data as in Figure 3 of the main text, here for R = 3.2 Å.

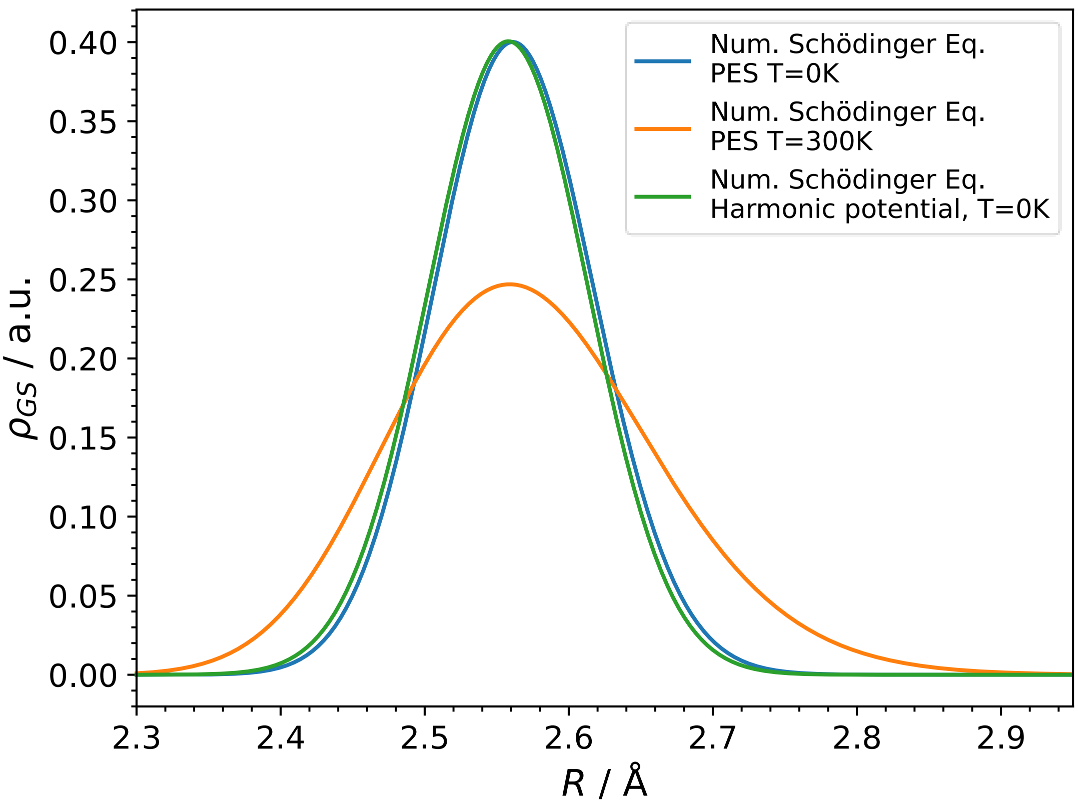

Figure S4: Comparison of the Zn_2^+ ground state density when solving the vibrational Schrödinger equation numerically for the vibrational ground state and for an average over vibrational states with the relative population determined by Boltzmann averaging at T = 0 K and T = 300 K (see Methods). Using the splined PES Calculated at the CCSD(T)(full)/aug-cc-pVQZ level as well as a harmonic potential of the vibrational frequency 169.71 cm^{-1} obtained at the same level of theory at a minimum distance of 2.558 Å.

Table S1: Zn–Zn distance R (in Å), excitation energies E (in eV) and oscillator strengths f in Zn_2^+ at various levels of theory.

Method	Basis	R_{min}	f (R = R_{min})	R (f = f_{min})	E (f = f_{min})	f (R = 2.68 Å)	E (R = 2.68 Å)	f (R = 2.48 Å)	E (R = 2.48 Å)	f (R = 2.96 Å)	E (R = 2.96 Å)
EOMCCSD	aug-cc-pVDZ	2.609	6.30E-03	2.71	2.57	4E-04	2.61	0.036	2.83	0.031	2.20
EOMCCSD / full	aug-cc-pVDZ	2.543	1.74E-02	2.70	2.60	2E-04	2.63	0.035	2.86	0.034	2.22
EOMCCSD	aug-cc-pVTZ	2.603	6.80E-03	2.70	2.59	2E-04	2.62	0.036	2.84	0.033	2.21
EOMCCSD / full	aug-cc-pVTZ	2.591	7.70E-03	2.69	2.63	1E-04	2.64	0.034	2.87	0.036	2.23
EOMCCSD	aug-cc-pVQZ	2.596	8.70E-03	2.71	2.58	4E-04	2.62	0.038	2.85	0.032	2.21
EOMCCSD / full	aug-cc-pVQZ	2.581	1.02E-02	2.69	2.64	2E-04	2.65	0.036	2.88	0.035	2.24
EOMCCSD	aug-cc-pWCVTZ	2.600	6.20E-03	2.68	2.64	0.0000	2.64	0.034	2.87	0.037	2.23
MRCI(3,5)	aug-cc-pVDZ	2.621	1.30E-02	2.78	2.37	5E-03	2.50	0.047	2.71	0.011	2.13
MRCI(3,5)	aug-cc-pVTZ	2.619	1.36E-02	2.79	2.36	5E-03	2.50	0.047	2.71	0.011	2.13
MRCI(3,5)	aug-cc-pVQZ	2.613	1.55E-02	2.80	2.35	6E-03	2.50	0.049	2.71	0.010	2.13
MRCI(3,9)	aug-cc-pVDZ	2.636	8.07E-03	2.76	2.43	3E-03	2.53	0.044	2.76	0.017	2.15
MRCI(3,9)	aug-cc-pVTZ	2.634	8.53E-03	2.76	2.43	3E-03	2.53	0.045	2.76	0.017	2.14
MRCI(3,9)	aug-cc-pVQZ	2.628	1.02E-02	2.76	2.43	4E-03	2.53	0.047	2.76	0.016	2.15
MRCI(3,11)	aug-cc-pVDZ	2.625	1.46E-02	2.80	2.38	6E-03	2.54	0.055	2.76	0.013	2.15
MRCI(3,11)	aug-cc-pVTZ	2.621	1.60E-02	2.79	2.40	6E-03	2.54	0.056	2.76	0.013	2.15
MRCI(3,11)	aug-cc-pVQZ	2.616	1.88E-02	2.80	2.38	8E-03	2.54	0.059	2.75	0.011	2.16
CAM-B3LYP	aug-cc-pVTZ	2.587	3.00E-03	2.65	2.98	9E-04	2.93	0.024	3.15	0.053	2.56

Table S2: Minimum distance R_{min} (in Å) and energies E (in eV) in Zn_2^+ at different distances at various levels of theory.

Method	Basis	R_{min}	$E(R = 2.7$ Å)	$E(R = 2.5$ Å)	$E(R = 3.2$ Å)
CCSD	aug-cc-pVDZ	2.609	0.012	0.022	0.306
CCSD / full	aug-cc-pVDZ	2.543	0.040	0.004	0.425
CCSD	aug-cc-pVTZ	2.603	0.013	0.019	0.306
CCSD / full	aug-cc-pVTZ	2.591	0.017	0.015	0.322
CCSD	aug-cc-pVQZ	2.596	0.016	0.017	0.317
CCSD / full	aug-cc-pVQZ	2.581	0.020	0.012	0.329
CCSD(T)	aug-cc-pVDZ	2.591	0.017	0.016	0.328
CCSD(T) / full	aug-cc-pVDZ	2.526	0.050	0.001	0.452
CCSD(T)	aug-cc-pVTZ	2.584	0.019	0.013	0.329
CCSD(T) / full	aug-cc-pVTZ	2.570	0.025	0.009	0.348
CCSD(T)	aug-cc-pVQZ	2.576	0.022	0.011	0.343
CCSD(T) / full	aug-cc-pVQZ	2.558	0.029	0.006	0.359

Table S3: Excitation energies E and oscillator strengths f of the first excited states in the energy minimum at 2.581 Å at CCSD(full)/aug-cc-pVQZ level. Calculated at the EOM-CCSD(full)/aug-cc-pVQZ level of theory.

State	E / eV	f
$A^2\Sigma_g^+$	2.77	0.0102
$B^2\Pi_u$	3.26	0.0000
$C^2\Sigma_g^+$	4.53	0.8757
$D^2\Pi_g$	4.88	0.0324
$E^2\Sigma_u^+$	5.41	0.0000

Experimental points from Figure 1

Energy / eV	Signal / cm^{-2}	Detection Limit cm^{-2}
5.5103	1.46E-19	6.57E-20
5.486	2.21E-19	7.67E-20
5.4618	0	5.90E-20
5.4378	1.90E-19	8.08E-20
5.4141	2.67E-19	9.05E-20
5.3905	3.14E-19	1.08E-19
5.3672	1.37E-19	1.16E-19
5.3441	2.27E-19	1.45E-19
5.3211	0	1.35E-19
5.2984	0	1.53E-19
5.2759	0	1.33E-19
5.2535	0	1.41E-19
5.2313	0	1.48E-19
5.2094	2.98E-19	1.83E-19
5.1876	2.82E-19	1.84E-19
5.1659	0	1.93E-19
5.1445	2.03E-19	1.35E-19
5.1232	2.23E-19	1.65E-19
5.1022	1.99E-19	1.40E-19
5.0813	2.64E-19	1.80E-19
5.0605	0	1.52E-19
5.0399	0	1.82E-19
5.0195	0	1.75E-19
4.9993	0	1.90E-19
4.9792	0	1.78E-19
4.9593	0	7.43E-20
4.9395	0	7.59E-20
4.9199	0	7.51E-20
4.9005	0	6.60E-20
4.8812	0	7.43E-20
4.8621	8.71E-20	5.27E-20
4.8431	1.52E-19	7.80E-20
4.8242	7.45E-20	7.40E-20
4.8055	1.54E-19	8.42E-20
4.787	1.42E-19	6.74E-20
4.7686	1.36E-19	7.41E-20
4.7503	1.11E-19	8.72E-20
4.7322	1.63E-19	7.94E-20
4.7142	1.94E-19	6.96E-20
4.6963	2.44E-19	9.01E-20
4.6786	1.98E-19	8.60E-20
4.661	1.14E-19	6.84E-20
4.6435	5.67E-20	5.05E-20
4.6262	0	5.99E-20
4.609	0	5.47E-20

Energy / eV	Signal / cm^{-2}	Detection Limit cm^{-2}
4.5919	0	5.29E-20
4.575	0	6.59E-20
4.5582	7.45E-20	5.36E-20
4.5415	1.03E-19	5.39E-20
4.5249	0	7.20E-20
4.5085	0	5.55E-20
4.4921	0	6.14E-20
4.4759	0	7.23E-20
4.4598	0	8.63E-20
4.4438	0	7.25E-20
4.428	0	7.64E-20
4.4122	0	8.40E-20
4.3965	0	7.20E-20
4.381	0	7.56E-20
4.3656	0	6.17E-20
4.3503	0	7.79E-20
4.3351	0	8.81E-20
4.32	0	1.04E-19
4.305	0	8.77E-20
4.2901	0	1.39E-19
4.2753	0	1.45E-19
4.2606	0	1.06E-19
4.246	0	8.78E-20
4.2315	0	8.49E-20
4.2171	0	8.85E-20
4.2028	0	8.14E-20
4.1328	2.72E-20	1.09E-20
4.1054	3.90E-20	1.19E-20
4.0784	3.77E-20	1.09E-20
4.0517	1.25E-20	1.06E-20
4.0254	1.90E-20	1.11E-20
3.9994	4.38E-20	1.19E-20
3.9738	1.45E-20	9.40E-21
3.9485	1.36E-20	9.57E-21
3.9235	1.77E-20	1.06E-20
3.8988	2.83E-20	1.07E-20
3.8745	1.99E-20	1.12E-20
3.8504	3.21E-20	9.91E-21
3.8266	2.06E-20	8.06E-21
3.7915	1.45E-20	8.42E-21
3.757	2.71E-20	8.24E-21
3.7232	2.43E-20	7.75E-21
3.69	0	8.05E-21
3.6573	0	8.26E-21
3.5937	4.15E-20	6.83E-21
3.5424	8.28E-21	6.54E-21
3.4925	7.23E-21	6.56E-21
3.444	1.82E-20	7.60E-21

Energy / eV	Signal / cm^{-2}	Detection Limit cm^{-2}
3.3968	2.60E-20	7.82E-21
3.3509	1.63E-20	7.96E-21
3.3062	9.66E-21	8.72E-21
3.2627	1.39E-20	7.63E-21
3.2203	9.63E-21	6.24E-21
3.179	1.01E-20	6.99E-21
3.1388	1.48E-20	7.01E-21
3.0996	4.42E-20	1.03E-20
3.0613	6.99E-20	1.26E-20
3.024	4.68E-19	2.44E-21
3.024	1.08E-18	5.31E-21
2.9947	1.74E-18	5.28E-21
2.9661	2.10E-18	6.41E-21
2.938	2.67E-18	4.53E-21
2.9104	2.70E-18	4.71E-21
2.8833	2.61E-18	4.07E-21
2.8567	1.68E-18	5.18E-21
2.8307	1.05E-18	4.33E-21
2.805	4.78E-19	4.54E-21
2.7799	2.25E-19	4.46E-21
2.7552	6.51E-20	3.86E-21
2.7309	4.65E-21	4.18E-21
2.707	1.64E-20	3.07E-21
2.6836	6.47E-20	3.73E-21
2.6606	1.31E-19	4.33E-21
2.6379	2.22E-19	4.15E-21
2.6157	2.47E-19	3.94E-21
2.5938	2.80E-19	4.27E-21
2.5723	2.61E-19	4.56E-21
2.5511	3.02E-19	3.61E-21
2.5303	2.31E-19	4.47E-21
2.5098	1.85E-19	4.50E-21
2.4896	1.49E-19	4.19E-21
2.4698	1.36E-19	3.35E-21
2.4502	1.34E-19	3.49E-21
2.431	9.65E-20	4.29E-21
2.4121	7.97E-20	3.52E-21
2.3935	6.81E-20	3.83E-21
2.3751	5.74E-20	4.19E-21
2.3571	5.01E-20	4.27E-21
2.3393	3.74E-20	4.21E-21
2.3218	2.65E-20	4.17E-21
2.3045	2.09E-20	3.56E-21
2.2875	2.09E-20	3.65E-21
2.2707	1.78E-20	4.07E-21
2.2542	1.68E-20	4.93E-21
2.238	1.34E-20	3.80E-21
2.2219	8.47E-21	4.07E-21

Energy / eV	Signal / cm^{-2}	Detection Limit cm^{-2}
2.2061	1.09E-20	3.41E-21
2.1905	7.84E-21	4.24E-21
2.1751	9.02E-21	4.14E-21
2.1376	7.65E-21	5.48E-21
2.1014	7.39E-21	4.34E-21
2.0664	0	4.78E-21
2.0325	5.76E-21	4.96E-21
1.9997	0	4.68E-21
1.968	6.80E-21	5.50E-21
1.9372	0	5.44E-21
1.9074	0	6.11E-21

References

(1) Ončák, M.; Šištík, L.; Slavíček, P. Can theory quantitatively model stratospheric photolysis? Ab initio estimate of absolute absorption cross sections of ClOOCl. *J. Chem. Phys.* **2010**, *133* (17), 174303. DOI: 10.1063/1.3499599.
